# Gender-Specific Dietary and Lifestyle Patterns Associated with Cardiometabolic Risk: A Cross-Sectional Analysis

**DOI:** 10.3390/nu17101705

**Published:** 2025-05-17

**Authors:** Mauro Lombardo, Jesse C. Krakauer, Nir Y. Krakauer, Massimiliano Caprio, Andrea Armani, Alessandra Feraco

**Affiliations:** 1 Department for the Promotion of Human Science and Quality of Life, San Raffaele Open University, Via di Val Cannuta, 247, 00166 Rome, Italy; massimiliano.caprio@uniroma5.it (M.C.); andrea.armani@uniroma5.it (A.A.); alessandra.feraco@uniroma5.it (A.F.); 2 Corewell Health William Beaumont University Hospital, Royal Oak, MI 48073, USA; jckrakauer@gmail.com; 3 Department of Civil Engineering, City College of New York, New York, NY 10031, USA; nkrakauer@ccny.cuny.edu; 4 Laboratory of Cardiovascular Endocrinology, San Raffaele Research Institute, IRCCS San Raffaele Roma, Via di Val Cannuta, 247, 00166 Rome, Italy

**Keywords:** abdominal adiposity, plant-based diet, physical activity, zABSI, gender differences, cardiometabolic risk, sport classification

## Abstract

Background: Gender differences in dietary patterns and lifestyle behaviours may influence abdominal adiposity and cardiometabolic risk, but comprehensive analyses integrating these factors remain limited. Methods: We conducted a cross-sectional study including 1631 adults recruited from a centre specialising in nutrition and metabolic health. Food intake was assessed by 7-day food diaries and lifestyle behaviours were assessed by structured questionnaires. *Z* scores of a body shape index (zABSI) were calculated as a marker of abdominal adiposity. zABSI represents the standardised value of ABSI, an index specifically designed to assess abdominal adiposity independently of BMI Multivariable linear regression models, stratified by sex and adjusted for age, examined associations between dietary patterns, physical activity and zABSI. Results: Higher intake of plant-based protein was significantly associated with lower zABSI values in women (β = −0.052, *p =* 0.0053) but not in men (β = −0.015, *p =* 0.2675). Stratified analyses revealed that women in the middle tertile of plant-based protein intake showed significantly lower zABSI values than men. Combined analyses showed that women classified as physically active and high consumers of plant-based protein had the most favourable abdominal adiposity profiles (*p =* 0.0036). Participation in endurance and strength sports was associated with lower zABSI values in both sexes, whereas women engaged in team sports had the lowest zABSI values. No significant interaction terms between sex and lifestyle were identified; however, male sex remained an independent predictor of higher zABSI values. Conclusions: In this cross-sectional study, plant-based dietary patterns and physical activity were associated with lower abdominal adiposity, especially among women. These findings suggest the importance of gender-specific strategies to address cardiometabolic risk and emphasise the need for prospective studies to confirm these associations and clarify the underlying mechanisms.

## 1. Introduction

Cardiometabolic diseases represent a major cause of morbidity and mortality worldwide, and increasing evidence points to the role of modifiable lifestyle factors in defining individual risk profiles [1,2]. Among these factors, dietary habits, physical activity levels, smoking and sleep quality have been identified as major determinants of metabolic health [1,3,4].

Dietary patterns rich in plant-based foods and characterised by low consumption of processed meats have been associated with favourable cardiometabolic outcomes, including lower rates of obesity, type 2 diabetes and cardiovascular disease [5,6,7]. In contrast, sedentary lifestyles, smoking and poor sleep quality were independently linked to increased abdominal adiposity and metabolic dysfunction [8].

Thus, evidence suggests the existence of gender differences in dietary choices, lifestyle behaviours and subsequent development of cardiometabolic risk [9,10]. Women generally report higher consumption of fruits, vegetables and plant-based proteins, whereas men tend to consume greater amounts of red and processed meats [9,11,12]. Physical activity patterns and smoking prevalence also vary between the sexes, potentially contributing to gender-specific health trajectories [10,13]. Large-scale cohort studies have also outlined the need for sex-specific analyses in nutritional epidemiology, given the different dietary patterns and metabolic responses observed in men and women [14,15].

Despite the increasing recognition of these differences, few studies have comprehensively explored how combined dietary and lifestyle behaviours are associated with cardiometabolic risk indicators in a gender-specific manner. In particular, body shape indicators such as a body shape index (ABSI), which captures abdominal adiposity independent of body mass index (BMI), offer valuable insights for early metabolic risk stratification [16,17]. Unlike BMI, which does not account for fat distribution, ABSI incorporates waist circumference and provides a more specific estimation of central adiposity, which is closely linked to cardiometabolic risk.

The aim of the present study was to examine gender-specific associations between food preferences, lifestyle patterns and the ABSI *z* score (zABSI) in a cohort of adults assessed for nutrition and metabolic health. Particular attention was given to the influence of plant-based protein intake, physical activity, smoking habits and sleep quality on body composition indicators.

## 2. Methods

### 2.1. Participants

A cross-sectional study was conducted between January and December 2024 at a specialised centre in Rome, Italy, focusing on nutrition and metabolic health. Recruitment included people attending clinical counselling centres and responding to targeted advertisements. Inclusion criteria were age between 18 and 75 years, knowledge of Italian sufficient to complete an online survey, and informed consent. Exclusion criteria included pregnancy, breastfeeding, treatment with drugs known to affect body weight (such as glucocorticoids, oestrogen or anticonvulsants) and diagnosis of chronic conditions such as alcoholism, kidney disease or diabetes mellitus. Participants were also excluded if they had incomplete datasets or if their declared energy intake, based on food diaries, was lower than their estimated basal metabolic rate. After applying these criteria, out of 1800 initial participants, 1631 persons were included in the analysis. The study population, recruited in a specialised clinical setting, may differ from the general population in terms of increased awareness of body composition and lifestyle management. However, the aim was to collect unbiased information on dietary habits, physical activity and body composition, regardless of initial health motivations. The study protocol, including informed consent procedures, was approved by the Ethics Committee of the IRCCS San Raffaele in Rome (registration number RP 23/13) and conducted in accordance with the Declaration of Helsinki.

### 2.2. Investigation

Food intake was assessed using 7-day food diaries, focusing on protein-rich food sources such as meat, processed meat, fish, eggs, dairy products, legumes and soy foods. To improve accuracy, participants received standardised instructions on portion estimation, supported by visual material and practical examples. Registered dieticians manually examined each diary to quantify the weekly frequencies and portion sizes of the reported foods. Participants who did not report intake of a specific food were classified as non-consumers, while consumers were stratified into low- and high-intake groups based on median intake values among consumers. These data were used to construct the Plant-Based Protein Score and the Mediterranean Model Score according to predefined criteria. Previous validation studies, including those of Brunner et al. and the Malmö Food Study [18,19], support the use of 7-day food diaries as a reasonably reliable method of assessing macronutrient intake compared to biomarkers and weighted food records. Prior to the clinical visit, participants completed a structured online survey accessible via any Internet-enabled device. The questionnaire, lasting approximately 30 min, collected detailed information on food preferences, frequency of consumption of food groups, interest and engagement in physical activity, smoking habits, sleep quality and other lifestyle behaviours potentially associated with cardiometabolic risk. Specific sections were designed to assess regularity of exercise practice, self-reported smoking status and perceived sleep patterns. Participants were asked to indicate the type of sport they regularly practised. The reported activities were then classified into four groups (endurance, skill, strength training and team sports) according to physiological demand and training characteristics, following a predefined classification scheme (see Supplementary Table S1). These categories were used to analyse associations with the zABSI. This comprehensive framework allowed for an in-depth assessment of gender-specific dietary and lifestyle profiles. Although the lifestyle questionnaire was not formally validated, this limitation should be considered when interpreting the findings. Nonetheless, its design followed commonly used formats in nutritional epidemiology [20,21]. Participation was anonymous and voluntary, with consent obtained electronically at the start of the survey.

### 2.3. Body Composition

Anthropometric and body composition measurements were performed following standardised procedures to minimise variability. Participants came to the clinic in the morning after fasting for at least three hours and wore only light undergarments during the assessments. Body weight was measured with a calibrated electronic scale (Tanita BC-420 MA, Tokyo, Japan) with an accuracy of 100 g. Measurements were taken on a stable surface, with participants standing barefoot. Two readings were obtained, with a third measurement if discrepancies exceeded 100 g; the two closest values were averaged. Standing height was measured using a stationary stadiometer, with participants aligned vertically (heels, buttocks, shoulders and head) and positioned on the Frankfurt plane. Again, two readings were taken and a third if differences greater than 0.1 cm were observed. BMI was calculated as weight (kg) divided by height squared (m^2^). Waist circumference was measured at the midpoint between the lower edge of the ribs and the iliac crest with participants standing, relaxed and breathing normally. Body composition parameters, including fat mass (FM) and fat-free mass (FFM), were assessed using bioelectrical impedance analysis (BIA) with the Tanita BC-420 MA device, validated by air displacement plethysmography (BodPod) [22]. Participants were instructed to avoid strenuous physical activity, excessive food intake, alcohol and caffeine for at least 12 h before the measurement. Female participants were encouraged to schedule assessments outside the menstrual phase to minimise the effects of fluid retention.

### 2.4. Dietary Pattern Scores and Calculation of ABSI

Weekly food intake frequencies were collected from dietary records obtained at the first follow-up after nutritional counselling. The Plant-Based Protein Score was calculated by summing the weekly consumption frequencies of legumes and soy products. The Mediterranean Model Score, ranging from 0 to 6, was developed to reflect adherence to a Mediterranean-type protein intake. One point was assigned for each of the following criteria: consumption of legumes ≥ 3 times/week, fish ≥ 2 times/week, eggs ≥ 3 times/week, dairy products between 2 and 6 times/week, total meat (including processed meat) ≤ 3 times/week and soy ≥ 1 time/week. This approach is based on previous scoring methods used in nutritional epidemiology and adapted to the available dietary data. A healthy protein score was also derived, defined as the sum of weekly intake of legumes and fish minus the frequency of processed meat consumption. ABSI was calculated according to the method proposed by Krakauer and Krakauer [16], using the following formula:

ABSI={WC}{BMI{2/3}Height{1/2}}

Waist circumference (WC) and height were measured in metres. BMI was calculated in kg/m^2^. The ABSI values were then standardised into *z* scores (zABSI) within the study population by subtracting the sample mean and dividing by the sample standard deviation. As expected for standardised variables, zABSI values are expressed in standard deviation units and have a mean close to zero. Due to the skewed distribution of ABSI in the sample, the standard deviation exceeds the absolute value of the mean. This internal standardisation allows for the interpretation of abdominal adiposity relative to the distribution within the study sample. Since ABSI is a dimensionless index derived from the ratio of these components, it has no units. It reflects qualitative changes in body shape and abdominal volume, rather than representing an absolute anthropometric measurement.

### 2.5. Statistical Analysis

The continuous variables were subjected to a normality test using the Shapiro–Wilk test. Since several variables were not normally distributed, non-parametric tests were applied. Gender differences were assessed using the Mann–Whitney U-test for continuous variables and the chi-square test for categorical variables. Associations between lifestyle factors and zABSI were analysed using multivariable linear regression models. Separate models were constructed to assess the main effects and to investigate potential gender-specific interactions by including sex-variable interaction terms. The Plant-Based Protein Score and Mediterranean Model Score were analysed as continuous, categorical variables, using the median hypothesis to define cut-offs. Differences in zABSI between multiple groups (e.g., sport categories, combined diet and physical activity profiles) were assessed using the Kruskal–Wallis tests. All models were adjusted for age. A two-tailed *p*-value < 0.05 was considered statistically significant. Statistical analyses were conducted with SPSS version 28.0 (IBM Corp., Armonk, NY, USA).

## 3. Results

At baseline, males were younger (mean ± SD, 40.42 ± 13.37 years) than females (42.30 ± 13.55 years; *p =* 0.0054) and had higher values of weight, height, BMI, ABSI and fat mass, all statistically significant at *p <* 0.001. Females, on the other hand, showed higher percentages of fat mass. No significant differences between genders were found for the Plant-Based Protein Score (*p =* 0.2198) or the Mediterranean Model Score (*p =* 0.0724). All body composition measures, dietary scores and anthropometric indices are summarised in Table 1.

**Figure 1:**
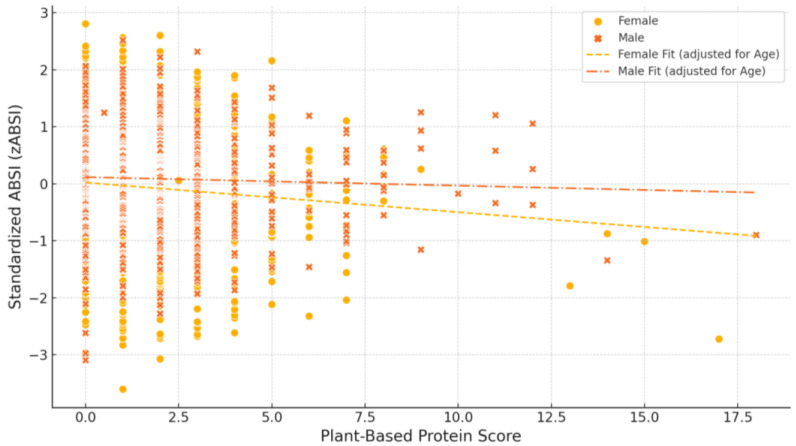
Association between plant-based protein intake and standardised ABSI (zABSI) by gender. The figure illustrates the association between Plant-Based Protein Score and standardised ABSI (zABSI) in male and female participants, adjusted for age using linear regression models. Scatter plots represent individual data points, while adjusted regression lines are shown for each sex according to the age-adjusted models for the respective group. Among females, a significant inverse association was observed between Plant-Based Protein Score and zABSI (β = −0.052, *p =* 0.0053), whereas no significant association was found among males (β = −0.015, *p =* 0.2675).

After adjustment for age, a significant inverse association was observed between the Plant-Based Protein Score and the zABSI in female participants (β = −0.052, *p =* 0.0053). No significant association was found among male participants (β = −0.015, *p =* 0.2675) (Figure 1).

**Figure 2:**
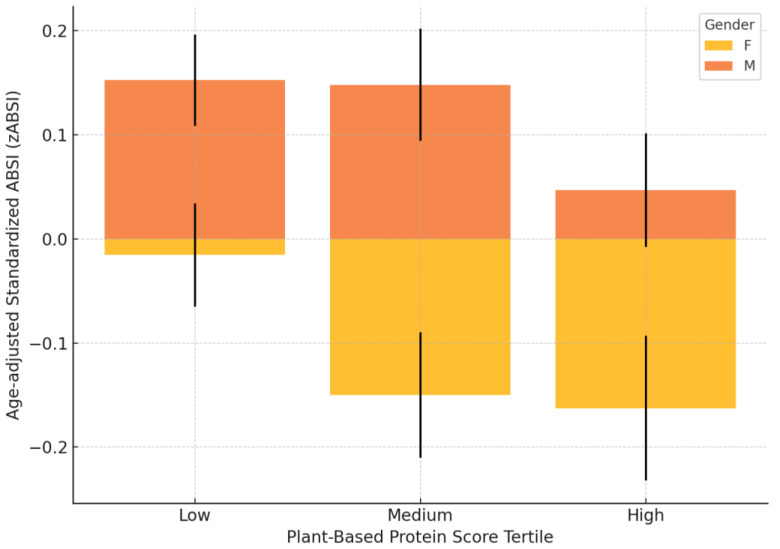
zABSI by Plant-Based Protein Score tertiles. Age-adjusted standardised ABSI values (zABSI) according to Plant-Based Protein Score tertiles and gender. The bars represent the mean ± standard error of the mean (SEM). A significant gender difference was observed in the middle tertile group (*p =* 0.010).

Among participants stratified according to Plant-Based Protein Score tertiles and after adjustment for age, a significant gender difference in zABSI was identified in the medium-intake group, where females had lower zABSI values than males (females: −0.15 ± 0.06, *n =* 251; males: 0.15 ± 0.05, *n =* 174; *p =* 0.010). No significant differences were found in the low (females: −0.02 ± 0.05, *n =* 445; males: 0.15 ± 0.04, *n =* 325; *p =* 0.152) or high (females: −0.16 ± 0.07, *n =* 244; males: 0.14 ± 0.06, *n =* 158; *p =* 0.215) intake groups (Figure 2).

**Figure 3:**
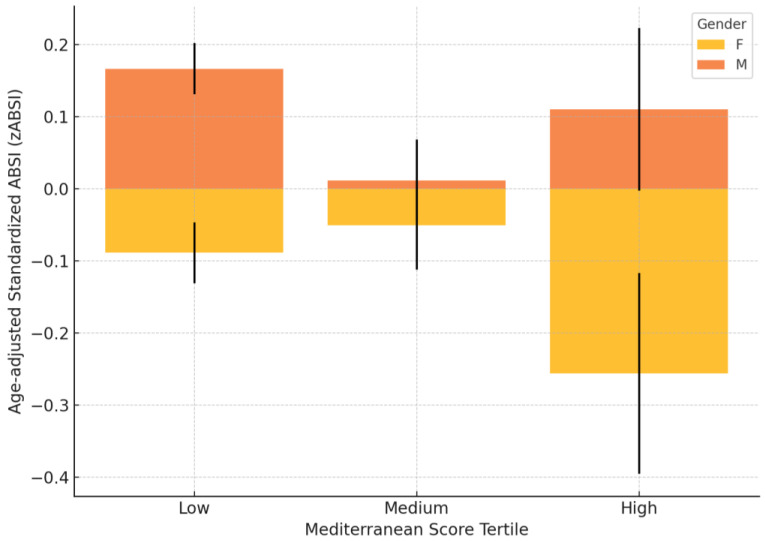
zABSI by mediterranean-score tertiles. Age-adjusted standardised ABSI (zABSI) values by Mediterranean Model Score tertiles and gender. The bars represent the mean ± standard error of the mean (SEM). A significant difference between the sexes was observed in the low tertile group (*p =* 0.003).

Among the participants stratified according to Mediterranean Model Score tertiles and after adjustment for age (Figure 3), a significant gender difference in the zABSI was identified in the low-intake group, where females had lower zABSI values than males (females: −0.09 ± 0.04, *n =* 598; males: 0.17 ± 0.04, *n =* 464; *p =* 0.003). No significant differences were found in the medium (females: −0.05 ± 0.06, *n =* 277; males: 0.01 ± 0.06, *n =* 184; *p =* 0.715) or high (females: −0.26 ± 0.14, *n =* 66; males: −0.04 ± 0.12, *n =* 50; *p =* 0.285) adherence groups.

**Figure 4:**
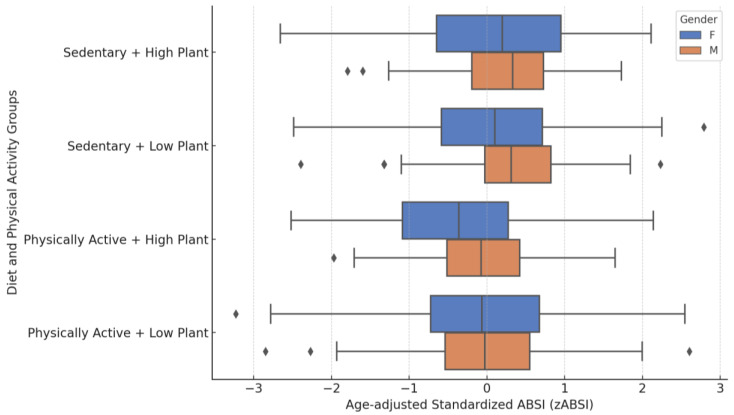
zABSI by diet and physical activity group and gender. Age-adjusted standardised ABSI (zABSI) according to the combined categories of plant protein intake and physical activity, stratified by gender. Females showed significantly lower zABSI values than males in the Physically Active + High Vegetarian, Sedentary + Low Vegetarian and Sedentary + High Vegetarian groups. The differences in zABSI between the combined groups were highly significant (Kruskal–Wallis *p <* 0.0001).

After adjustment for age, gender-specific differences in zABSI were observed in the combined diet and physical activity groups (Figure 4). Females had significantly lower zABSI values than males in the Physically Active + High Plants group (*p =* 0.0036), the Sedentary + Low Plants group (*p =* 0.0083) and the Sedentary + High Plants group (*p =* 0.0297). No significant difference between the sexes was found in the Physically Active + Low Plants group (*p =* 0.896). The overall comparison between the four groups combined showed highly significant differences in zABSI (Kruskal–Wallis H = 76.5, *p <* 0.0001).

**Figure 5:**
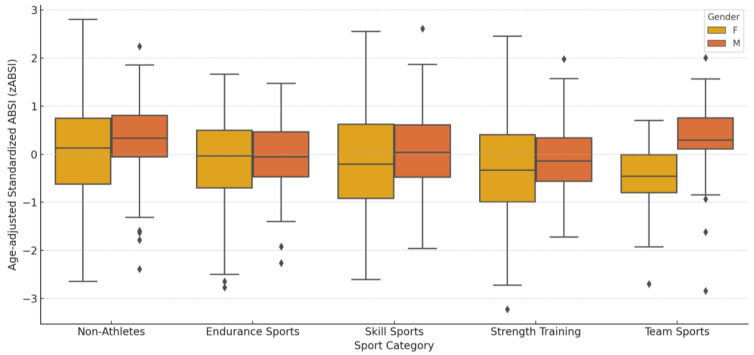
Gender differences in zABSI across sport groups. Age-adjusted standardised ABSI (zABSI) by sport category and gender. Bars represent median and interquartile range. A significant difference between the sexes was observed in the non-athletes and team sports groups, with females showing lower zABSI values than males.

After adjustment for age, non-athletes presented the highest zABSI values among both males and females (Figure 5). Females showed significantly lower zABSI values than males in the non-athlete group (*p <* 0.0001) and in the team sports group (*p =* 0.0012). No significant gender differences were observed in the Endurance (*p =* 0.330), Skill (*p =* 0.141) or Strength Training (*p =* 0.889) groups. Overall, the Kruskal–Wallis test confirmed highly significant differences in zABSI between the sport categories (*p <* 0.0001).

A summary of the mean values of zABSI by sport category and gender is shown in Supplementary Table S2. Non-athletes presented the highest zABSI values, especially among males. Women engaged in skill sports and strength training showed lower zABSI values than their male counterparts.

To specifically investigate gender differences in the effects of lifestyle factors on the zABSI, interaction terms between gender and each variable were included in the multivariable model. As shown in Table 2, no significant interactions were found. This indicates that, although men had generally higher zABSI values, the associations of plant-based protein intake, physical activity, smoking and sleep quality with abdominal adiposity were comparable in direction and magnitude between the two sexes.

## 4. Discussion

Our results suggest a sex-specific association between plant protein intake and abdominal adiposity as assessed by the zABSI. Specifically, a higher intake of plant-based protein was associated with lower zABSI values in women, but not in men. In general, previous evidence documents the benefits of plant-based versus animal-based protein in terms of adiposity reduction and metabolic improvement [23,24]. A sex-differential association may indicate physiological mechanisms, including variations in hormonal milieu, fat distribution patterns and gut microbiota composition, which have been shown to modulate the metabolic effects of dietary components [25,26]. However, in large cohort studies, the effects of abdominal adiposity and adherence to plant-based diets showed only a few differences between women and men [27,28]. The lack of association between plant protein intake and zABSI observed in males could be attributed to lower intake of plant-based foods at baseline, differences in the quantity or quality of protein sources consumed or divergent lifestyle behaviours that could offset dietary effects [29,30].

Specific biological mechanisms are likely to contribute to a differential response of abdominal obesity to dietary patterns. Oestrogenic modulation of adipose tissue distribution, known to favour a gynoid pattern with lower visceral fat accumulation in premenopausal women, may interact with dietary components such as phytoestrogens present in plant-derived foods [31]. Furthermore, sex-related differences in the composition of the gut microbiota, recently identified as a mediator of energy homeostasis and adiposity [32], could contribute to the different metabolic responses observed. Taken together, these factors suggest that plant-derived proteins may exert greater anti-inflammatory and insulin-sensitising effects in women, potentially leading to more pronounced reductions in abdominal adiposity than in men [33,34,35].

Although our cross-sectional design precludes causal inferences, the observed associations between dietary and lifestyle behaviours and abdominal adiposity provide important insights into potential gender-specific strategies for cardiometabolic risk reduction. In particular, women with a higher adherence to plant-based protein intake and those classified in the healthiest physical activity categories showed lower zABSI values than their male counterparts. These patterns were also evident when examining adherence to a Mediterranean dietary pattern, although the association appeared less robust compared to the Plant-Based Protein Score. Overall, these results highlight the potential of diet quality and structured physical activity as key correlates of favourable body composition profiles, especially among women [36,37]. However, longitudinal and intervention studies are needed to clarify the causal pathways underlying these relationships and to better define sex-specific recommendations [38].

Physical activity patterns also played a key role in sex differences in abdominal adiposity. In line with previous studies reporting that sedentary behaviour is more strongly associated with visceral fat accumulation in men than in women [39], we found significantly higher zABSI values among inactive males than females. Participation in team sports may be associated with lower abdominal adiposity, especially in women, consistent with evidence that structured and socially engaging physical activities promote better metabolic outcomes in female populations [40,41]. Among endurance athletes and those engaged in strength training, no significant gender differences in zABSI emerged, suggesting that higher exercise volumes or intensities may compensate for biological disparities in fat distribution [42]. This aligns with evidence showing men improve fitness more with resistance training, while women tend to reduce central adiposity more effectively [43,44]. These findings support the development of gender-specific strategies for cardiometabolic prevention.

Consistent with these observations, stratified analyses further emphasised the interaction between diet, physical activity and sex differences in abdominal adiposity. In the middle tertile of plant-based protein intake, women showed significantly lower zABSI values than men, whereas no significant sex differences were observed in the lowest or highest tertiles. This non-linear trend may reflect a threshold effect, whereby a moderate but prolonged intake of plant-based protein is sufficient to influence the distribution of abdominal fat in women, whereas higher intakes may not yield additional benefits. Similarly, among participants with poor adherence to the Mediterranean dietary pattern, women had significantly lower zABSI values than men. In the combined analysis of diet and physical activity, women in the Physically Active + High Plant Protein group showed the most favourable body composition profiles, with significantly lower zABSI values than their male counterparts. These results are in line with previous research suggesting that adherence to multiple healthy lifestyle behaviours may have additive or synergistic benefits on abdominal fat distribution, particularly in women [45].

Sex-related differences in abdominal adiposity may be supported by multiple physiological mechanisms. Oestrogen plays a central role in regulating fat distribution, promoting subcutaneous fat deposition and limiting visceral fat accumulation [46]. After menopause, oestrogen deficiency is associated with a shift towards central adiposity and increased cardiometabolic risk [47]. In addition, women tend to expand fat reserves mainly through adipocyte hyperplasia, whereas men more commonly exhibit adipocyte hypertrophy, especially in the visceral depot, contributing to an increased metabolic risk [48]. These patterns are further influenced by sex-specific differences in androgen receptor expression and steroidogenic enzyme activity in adipose tissue [49]. In addition, oestrogen may exert site-specific effects on lipolysis, increasing the mobilisation of fat in intra-abdominal deposits but not in subcutaneous tissue [50]. The oestradiol–ERα signalling pathway has also been shown to reduce adipogenesis and autophagy, limiting visceral fat accumulation in premenopausal women [51]. Although evidence directly addressing sex-specific responses to plant-based protein intake is limited, differences in gut microbiota composition and oestrogen-sensitive metabolic pathways may contribute to divergent physiological responses between men and women. This remains a promising area for future mechanistic or stratified studies. Furthermore, in postmenopausal women, changes in SHBG and estrone levels influence lipid metabolism and are key mediators of the relationship between abdominal adiposity and cardiometabolic risk [52]. Even in subgroups where statistical significance was not achieved, the consistent directionality of the associations between dietary and lifestyle factors and abdominal adiposity may have biological relevance that merits further investigation.

With regard to sports participation, non-athletes of both sexes showed the highest zABSI values, although women maintained lower levels than men. Participation in endurance and strength sports may be associated with reduced zABSI values in both sexes, while women involved in team sports achieved the lowest zABSI values overall, in contrast to the highest values among male participants in team sports [43]. These data underline the role of sport type and engagement context in modulating central fat accumulation, potentially in a gender-specific manner [53,54]. Although no significant interaction terms between gender and lifestyle were identified in multivariable models, male gender remained an independent predictor of higher zABSI, which strengthens the concept of intrinsic biological influences on abdominal adiposity that persist beyond modifiable lifestyle factors [55].

Several limitations of this study must be recognised. Firstly, the cross-sectional design limits the ability to establish causal relationships between dietary and lifestyle behaviour and abdominal adiposity. Secondly, although food intake was assessed using 7-day food diaries reviewed by qualified dietitians, a certain degree of reporting bias and under- or overestimation of intake cannot be completely excluded. Third, data on lifestyle behaviours were collected using a structured but not validated questionnaire, which may introduce classification or reporting bias. Fourth, although the Mediterranean Model Score was explicitly defined and adapted from established criteria, it was not validated against widely used instruments such as the PREDIMED index, limiting comparability with other studies. Fifth, although the zABSI provides a refined estimate of central adiposity compared to conventional anthropometric indices, it remains an indirect surrogate and does not allow direct quantification of visceral fat. Sixth, the study population, recruited in a specialised clinical setting, may differ from the general population in terms of health awareness and behaviour, introducing a potential selection bias and limiting the external generalisability of the results. Seventh, the relatively small sample size—especially when assessing sex differences between subgroups—introduces uncertainty regarding the magnitude of some observed effects. Although age was adjusted as a continuous covariate in all models, we did not stratify by age groups (e.g., <50 vs. ≥50 years), which could have revealed further interactions between age, sex and abdominal adiposity. Future studies with larger sample sizes should consider stratified or interaction analyses to explore these effects. Finally, despite adjustment for the main confounding factors, the possibility of residual confounding by unmeasured variables cannot be excluded.

## 5. Conclusions

Our results show sex-specific associations between plant-based dietary patterns, physical activity behaviour and abdominal adiposity. Women may benefit more from a higher intake of plant-based protein and an active lifestyle in terms of central fat distribution. These results support the development of gender-tailored interventions to reduce cardiometabolic risk. Prospective and interventional studies are needed to confirm these associations and to clarify the underlying biological mechanisms. Given its ability to detect subtle differences in abdominal adiposity, zABSI could support cardiometabolic risk stratification in both research and clinical settings, particularly when combined with sex-specific assessments. A summary of the key findings is presented in Table 3.

## Figures and Tables

**Table 1:** Baseline characteristics of the study sample. Baseline characteristics are presented as mean values with standard deviations in brackets. The comparison between male and female participants was conducted using Welch’s *t*-test. Significant differences were found for age, weight, height, BMI, standardised ABSI (zABSI), fat mass, free fat mass and basal metabolic rate (BMR). No significant differences between the sexes were observed for the Plant-Based Protein Score or the Mediterranean Model Score.

Variable	Total	Male Mean (SD)	Female Mean (SD)	*p*-Value
n	1631	690	941	
Age (y)	41.5 (13.5)	40.42 (13.37)	42.30 (13.55)	0.0054
Weight (kg)	80.31 (17.76)	89.85 (17.29)	73.31 (14.55)	<0.001
Height (m)	1.68 (0.09)	1.76 (0.07)	1.63 (0.06)	<0.001
BMI (kg/m^2^)	28.24 (5.23)	28.93 (5.11)	27.73 (5.26)	<0.001
ABSI	0.08 (0.00)	0.08 (0.00)	0.08 (0.01)	0.0007
zABSI	−0.01 (1.0)	0.09 (0.86)	−0.08 (1.08)	0.0007
fat mass (kg)	25.25 (10.72)	23.48 (10.85)	26.55 (10.44)	<0.001
fat mass (%)	30.79 (9.05)	25.03 (7.54)	35.01 (7.62)	<0.001
FFM (kg)	52.36 (11.4)	63.11 (8.27)	44.48 (5.29)	<0.001
FFM (%)	65.82 (8.67)	71.28 (7.26)	61.81 (7.32)	<0.001
BMR	1660.24 (348.12)	1966.33 (277.96)	1434.20 (182.57)	<0.001
Plant-Based Protein Score	1.93 (1.96)	2.00 (2.14)	1.87 (1.81)	0.2198
Mediterranean Model Score	2.07 (1.01)	2.02 (1.00)	2.11 (1.01)	0.0724

**Table 2:** Interaction terms assessing gender differences in associations between lifestyle factors and standardised ABSI (zABSI). Terms of interaction between gender and lifestyle factors in the multivariable model predicting standardised ABSI (zABSI). Beta coefficients (β), 95% confidence intervals (CI) and p-values are reported. No significant interactions were identified, indicating similar effects between genders.

Interaction Term	Beta (β)	95% CI Lower	95% CI Upper	*p*-Value
Sex × Physical Activity	−0.013	−0.173	0.147	0.8728
Sex × Plant-Based Protein Intake	0.094	−0.077	0.266	0.2790
Sex × Smoker	−0.012	−0.241	0.216	0.9158
Sex × Disturbed Sleep	0.048	−0.133	0.229	0.6031

**Table 3:** Key findings from the study. This table summarises the principal messages derived from the cross-sectional analysis of sex-specific associations between dietary patterns, lifestyle behaviours and abdominal adiposity (zABSI).

Key Message	Details
zABSI is a useful marker of central adiposity	The standardised ABSI (zABSI) effectively captures abdominal fat distribution beyond BMI.
Sex differences in abdominal fat are evident	Women had significantly lower zABSI values than men, indicating less central adiposity.
Plant-based diets may benefit women more than men	Higher plant protein intake was associated with lower zABSI in women but not in men.
Physical activity may be linked to lower central adiposity	Active individuals, particularly women, had more favourable zABSI profiles.
Gender-tailored strategies may enhance cardiometabolic prevention	Findings support the design of sex-specific dietary and lifestyle interventions.
Limitations include use of non-validated tools for behavioural assessment	Lifestyle data were collected via structured but non-validated questionnaires, potentially affecting accuracy.

## Data Availability

The datasets used and/or analysed during the current study are available from the corresponding author upon reasonable request. All data will be shared in an anonymized format to ensure participant confidentiality.

## Supplementary Materials

The following supporting information can be downloaded at https://www.mdpi.com/article/10.3390/nu17101705/s1, Table S1: Classification of sports activities by category; Table S2: Age-adjusted standardised ABSI (zABSI) by sport category and gender; Table S3. Gender-specific distribution of age-adjusted zABSI values across Plant-Based Protein Score groups.
