# Supplementary material for: Gender-Specific Dietary and Lifestyle Patterns Associated with Cardiometabolic Risk: A Cross-Sectional Analysis
Source: Nutrients. 2025 May 17;17(10):1705. doi: 10.3390/nu17101705 (PMC12114336; doi:10.3390/nu17101705)
Supplement: Supplementary file 1 [file nutrients-17-01705-s001.zip › nutrients-3649664-supplementary.pdf]

Table S1. Classification of Sports Activities by Category.

Endurance Sports	Skill Sports	Strength Training	Team Sports
Acquagym	Boxing	Calisthenics	Acrobatic Dance
Cycling	Capoeira	Functional Bodybuilding	Basketball
Elliptical Bike	Golf	Home Work Out	Handball
Hydrobike	Gymnastics	Pilates	Hockey
Rowing	Horse Riding	Powerlifting	Rugby
Running	Martial arts	Pump	Soccer
Spinning	Padel	Weightlifting	Volleyball
Step	Ping Pong		Waterpolo
Total Body	Pole Dance		
Treadmill	Skating		
Trekking	Tai Qui		
Walking	Tennis		
	Yoga		

Classification of sports activities into groups: Endurance Sports, Skill Sports, Strength Training, and Team Sports.

Table S2. Age-Adjusted Standardized ABSI (zABSI) by Sport Category and Gender.

Sport Category	Females (Mean ± SEM)	Males (Mean ± SEM)
Non-Athletes	0.06 (0.04)	0.32 (0.04)
Endurance Sports	-0.21 (0.10)	-0.02 (0.07)

Skill Sports	-0.18 (0.09)	0.08 (0.08)
Strength Training	-0.30 (0.08)	-0.11 (0.05)
Team Sports	-0.55 (0.23)	0.30 (0.20)

Age-adjusted standardized ABSI (zABSI) values (mean ± standard error of the mean) by sport category and gender. Non-athletes exhibited the highest zABSI values, while participants engaged in endurance, skill, strength training, and team sports showed more favorable body shape profiles.

Table S3. Gender-specific distribution of age-adjusted zABSI values across Plant-Based Protein Score groups.

Gender	Plant-Based Protein Score Group	Mean zABSI	SD	n	95% CI Lower	95% CI Upper
F	high	-0.044	1.104	125	-0.238	0.149
F	low	0.097	0.966	491	0.012	0.183
F	medium	0.150	1.013	119	-0.032	0.332
F	zero	0.175	1.026	206	0.035	0.315
M	high	-0.214	0.867	104	-0.381	-0.048
M	low	-0.066	0.950	332	-0.169	0.036
M	medium	-0.279	0.915	87	-0.471	-0.087
M	zero	-0.164	1.113	167	-0.333	0.004

Age-adjusted zABSI values by gender and Plant-Based Protein Score group. Mean, standard deviation (SD), sample size (n), and 95% confidence intervals are reported for each tertile and for non-consumers, separately for men and women.
